# A Novel Mechanism of High Dose Radiation Sensitization by Metformin

**DOI:** 10.3389/fonc.2019.00247

**Published:** 2019-04-09

**Authors:** Stephen L. Brown, Andrew Kolozsvary, Derek M. Isrow, Karine Al Feghali, Karen Lapanowski, Kenneth A. Jenrow, Jae Ho Kim

**Affiliations:** ^1^Department of Radiation Oncology, Henry Ford Hospital, Detroit, MI, United States; ^2^Department of Psychology, Central Michigan University, Mount Pleasant, MI, United States

**Keywords:** metformin, radiation, cancer, metabolism, glucose, tumor blood flow

## Abstract

**Introduction:** Metformin, the most widely used treatment for diabetes, is lethal to cancer cells and increases in toxicity when used in combination with radiation. In addition to various molecular and metabolic mechanisms that have been previously proposed, the studies presented provide evidence of an additional, novel mechanism of sensitization following high dose radiotherapy; the magnitude of sensitization depends on the microenvironmental levels of glucose and oxygen which are in turn affected by high dose radiation.

**Methods:** Cancer cells (A549 and MCF7) were studied *in vitro* under various controlled conditions. Endpoints included clonogenic cell survival and ROS expression measured by DHE and DCFDA. CD1 nu/nu athymic mice implanted with A549 cells received metformin alone (200 mg/kg, i.p.), radiation alone (15 Gy) or a combination of metformin and radiation; the effect of treatment sequence on efficacy was assessed by tumor growth delay and histology. In a separate set of experiments, tumor blood flow was measured using a tracer clearance technique using SPECT after the administration of metformin alone, radiation alone and the combined treatment.

**Results:** *In vivo*, metformin provided equally effective tumor growth delay when given 24 h after radiation as when given 1 h or 4 h before radiation, an observation not previously reported and, in fact, unexpected based on published scientific literature. When drug followed radiation, the tumors were histologically characterized by massive cellular necrosis. *In vitro*, cancer cells when glucose depleted and/or hypoxic were preferentially killed by metformin, in a drug dose dependent manner. A549 cells exposed to 5.0 mM of metformin was reduced seven fold in survival when in a glucose deprived as compared to a low-glucose medium (0 vs. 1.0 g/L). Finally, using a SPECT detector to follow the washout of a radioactive tracer, it was shown that a high single dose of radiosurgery (15 Gy) could dramatically inhibit blood flow and presumably diminish glucose and oxygen.

**Discussion:** Insight into the best timing of drug and radiation administration is gained through an understanding of the mechanisms of interaction. A new mechanism of metformin sensitization by high dose radiation is proposed based on the blood flow, glucose and oxygen.

## Introduction

Metformin is an inhibitor of complex 1 in the respiratory chain, and is a widely used therapy to reduce insulin resistance in diabetic patients. It has also been described to have pleotropic effects including inhibiting mTOR kinase via AMPK. Metformin has recently received epidemiological attention for its anticancer properties and preclinical attention for its cytotoxicity against cancer stem cells (1–5) including radiosensitization (6–10). Preclinical reports suggest that metformin reduces tumor hypoxia and would be beneficial if administered prior to radiotherapy (7, 8). No published scientific evidence supports administering metformin after radiation to enhance radiosensitization by metformin.

Building on two of our previous experimental observations, we hypothesized that tumor radiosensitization could be achieved by a high dose of radiation followed by metformin administration. First, we made the unique observation that another mitochontrial inhibitor, arsenic trioxide (ATO) had potent anti-cancer properties (11) and provided strong radiosensitization when radiation proceeds ATO (12, 13). We shall show that our experience with metformin parallels that of ATO in that the mitochondrial inhibitor alone, either metformin or ATO, have a marginal cytotoxic effect on hypoxic tumors whereas combining radiation with a mitochondrial inhibitor, either metformin or ATO, have a dramatic cytotoxic effect.

Second, we documented that high doses of radiation cause a rapid decrease in tumor blood flow (and not normal tissue blood flow) (14) and presumably this is immediately followed by precipitous drops in oxygen and glucose which we hypothesized could increase killing by metformin. A report by Miskimins and Chan indicate that treatment of cancerous ovarian cells with metformin resulted in a quantitative increase in ROS levels as measured by MitoSOX and DHE fluorescence (11). Since metformin is believed to act on mitochondrial respiratory complex I (increasing electron leakage to form superoxide from oxygen), thus driving energy production away from respiration and toward direct glucose utilization, we set out to test whether metformin use would lead to a glucose concentration-dependent increase in intracellular ROS, yielding cytotoxicity and radiosensitization.

The results presented support a new paradigm to optimally combine metformin with high dose radiotherapy.

## Materials and Methods

### Cell Lines and Culture

Cell lines, A549 derived from a lung cancer patient, and MCF-7 from a breast cancer patient, were purchased from American Type Culture Collection, ATCC (Manassas, VA). Both cell lines were cultured in Dulbecco's modified eagle media, DMEM, supplemented with ~10% fetal bovine serum (FBS), and 1% penicillin and streptomycin. Both also form colonies and allow for clonogenic survival assay.

We examined the following three conditions: DMEM high glucose which contained 4.5 mg/ml glucose, independent of serum, DMEM low glucose which contained 1.0 mg/ml glucose, independent of serum. And, DMEM no glucose which contained 0.0 mg/ml glucose, independent of serum. DMEM is referred to as glucose “free,” however some glucose was actually present due to the FBS. The glucose concentration in the FBS used was 1.04 mg/ml. We calculate 0.0937 mg/ml glucose was present in the media under all conditions due to the addition of serum.

### Quantification of ROS Levels

ROS are widely evaluated in tissue culture using dihydroethidium (DHE) and 2′,7′-dichlorofluorescein diacetate (DCFDA) (15, 16). We examined both biomarkers and found DHE to be more reliably predictive of cytotoxicity following radiation exposure. Levels of DHE were measured using flow cytometry. Tissue culture flasks of cells in exponential growth phase were grown to approximately 70–80% confluence. Cell culture media was removed and replaced with fresh media containing variable concentrations of metformin and glucose. In some experiments, cells were irradiated and/or maintained under conditions of low oxygen (i.e., hypoxia). Cells were incubated with metformin and under conditions of varying glucose and/or hypoxia for fixed periods of time. Following incubation, sufficient DHE reagent was added to yield 10 μmol/L solutions. The flasks were allowed to incubate for 45 min before removing the media, washing twice with cold PBS, and trypsizing the monolayers to generate single cell suspensions. The cell solutions were centrifuged for 5 min at 1800RPM and the resulting cell plugs were redistributed in 2 ml of cold PBS. These single cell solutions were analyzed for DHE signal using a small laptop flow cytometer (BD Accuri™ C6 Plus System, Ann Arbor, MI).

### Quantification of Cellular Cytotoxicity by Clonogenic Assay

MCF-7 and A549 cells were used when growing in exponential phase (~70–80% confluence). Cells were trypsinized to generate single cell suspensions, counted, and plated in appropriate numbers, between 800 and 4,000 cells depending on the anticipated cytotoxicity of the treatment, with fresh media. Within hours, culture media was replaced with media containing variable concentrations of metformin and allowed to incubate for a specific duration. Some flasks were exposed to radiation. In studies of hypoxia, 5% CO_2_, 95% N_2_ flowed continuously into a separate chamber within the temperature and humidity controlled cell culture incubator following our previously published practice to produce radiobiological hypoxia (17). The cells were allowed to incubate for 10–14 days. Crystal violet dye (0.05% crystal violet, 20% methanol, 80% distilled water) was added to flasks for 10 min to stain the cells, the media and dye was carefully removed, flasks were dried overnight and the resulting colonies counted manually using a light to magnify the shadow of colonies projected to a screen.

### Animals

Mice were housed in an AAALAC-approved facility. Animal procedures were reviewed and approved by the Henry Ford Health System institutional animal care and use committee (IACUC). Studies were performed in accordance with the IACUC-approved protocol and in accordance with published recommendations for the proper use and care of the laboratory animals. For the tumor growth delay experiments, 19–20 g CD-1 nu/nu athymic mice (Charles Rivers Laboratories, Wilmington, MA) were implanted with A549 cells. After trypsinization, 2 x 10^6^ cells were resuspended in a volume of 0.1 ml of normal saline and injected intramuscularly into the right gastrocnemius muscle using a 26 gauge needle and 1 mL syringe. From tumor cell injection to the start of the study was approximately 3 weeks, when the tumors were on average 9.0 ± 0.5 mm in diameter. Following treatment with sham, drug, irradiation or combined drug and irradiation, mice were either followed for tumor growth (tumor sizes were measured 2 or 3 times per week), euthanized for tumor histology, or used for tracer clearance studies. At the completion of the study, 90 days or if the tumors reach 1 cm^3^ in volume, mice were killed humanely using CO_2_ inhalation, consistent with the recommendations of the Panel on Euthanasia of the American Veterinary Medical Association.

### Drug

Metformin (Sigma-Aldrich, St. Louis, MO) was used at a dose of 200 mg/kg either alone or combined with radiation. The various schedules of metformin relative to radiation exposure included 1 h before radiation, 4 h before radiation, 24 h after radiation, and 72 h after radiation.

### Radiation

A dedicated laboratory irradiator was used to expose cultured cells in T-25 flasks to radiation. The radiation source was a 5,000 Ci Cesium-137 J.L. Shepherd and Associates (San Fernando, CA). Radiation dosimetry was initially commissioned by the company and followed by quarterly calibration checks that include an electrometer, micro-thermo-luminescence microdosimeters or GAFchromic film. A dedicated animal facility irradiator was used to expose murine legs with tumors.

Mouse tumors were irradiated using a Faxitron CP160 x-ray machine (Faxitron X-Ray Corp., Wheeling, IL, USA) operating at 160 kV with a 0.8 mm filter to harden the beam (18). The mice were placed at shelf position 7 from the source such that the dose rate was approximately 1 Gy/min. Mice were anesthetized with 80 mg/kg ketamine and 8 mg/kg xylazine. Positioning the anesthetized mice radially, with their right legs extending into the center and the remainder of the animal shielded allowed up to 10 mouse tumors to be irradiated at one time. No cover was used on the jig such that only air separated the x-ray source from the tumor.

### Tumor Effects

The effect of timing and sequence of metformin on radiation-induced (15 Gy) tumor growth delay was evaluated using A549 grown in the hind legs of athymic mice. Six groups of mice were used: no treatment controls sham irradiated, metformin alone (200 mg/kg), metformin 1 or 4 h before radiation, metformin 24 or 72 h after radiation. The three tumor endpoints were tumor growth delay, histological examination and tracer clearance. For tumor growth delay studies, a caliper accurate to 0.1 mm was used to measure two orthogonal tumor dimensions, d and D, including overlying skin. Tumor size was calculated using (4π/3)(D/2)(d/2)^2^ ≈ Dd^2^/2, where D and d are orthogonal diameters.

### Histological Examination

Some mice were euthanized 1 day after the last treatment (drug or radiation) and the tumor examined histologically. In these cases, 90 min prior to euthanasia, pimonidazole hydrochloride (Hypoxyprobe, Burlington, MA) at a dose of 30 mg/mL in 0.9% sterile saline was injected into the tail vein of mice. In the studies involving pimonidazole administration, mice were euthanized by cervical dislocation by a skilled technician. Tumors were dissected and half the tumor snap frozen in 2-methylbutane cooled with dry ice to ~-60 to −70°C, and subsequent FITC-conjugated anti-pimonidazole primary antibody while the other half was immersed in 10% formalin for subsequent H&E staining.

Two additional histological studies were performed. In the first, A549 tumors implanted in the hind muscle of CD-1 nu/nu mice as previously described were irradiated with 15 Gy, which is a subcurative dose of radiation. Approximately 3 weeks later when tumors were regrowing slowly compared to untreated control tumors (perhaps a consequence of impaired blood flow or compromised tumor microenvironment), metformin (200 mg/kg) was administered and tumors were harvested 2 h later and prepared for histological examination. Pimonidazole, as described above was included in these studies for assessment of hypoxic tissue regions. For comparison, control tumors were harvested 2 h after unirradiated mice were administered metformin (200 mg/kg).

The second additional histological study involved intradermally grown A549 tumors which have impaired blood flow compared with tumors grown intramuscularly (19). Groups of mice received 15 Gy alone, metformin (200 mg/kg) alone, or combined radiation and drug, with metformin delivered 1 h after radiation. Tumors were removed at a specified time (1 h, 4 h, 24 h, or 72 h) after last treatment and examined histologically as described above. Pimonidazole was not included in the intradermally grown tumor studies.

All histological studies were repeated at least three separate times.

### Tracer Clearance—Blood Flow Changes

Changes in blood flow were assessed using a tracer clearance method previously described (20). Briefly, following a direct injection using a 29 gauge needle, the rate of clearance of a deposited tracer, volume ~10 μL, is measured as a function of time to reflect the rate of tissue blood flow. The tracer used was Na^99m^TcO_4_. Tracer kinetics were monitored using a nuclear medicine scintillation detector (Albira SPECT, Bruker, Billerica, MA). Each condition was performed twice.

### Statistics

The tumor growth data presented are from experiments with groups of mice numbering 4 or 5; experiments were repeated multiple times if the variation in tumor volume at the completion of the study was large (defined as a standard error of the means, SEM, >20% of the average tumor volume, T_ave_). Repeat experiments continued until the variation in tumor volume at the completion of the study was small (defined as SEM <20% of T_ave_). A significance of interaction between treatment groups was tested for tumor growth delay between treatment groups at specific times using the Student's *t*-test.

## Results

### Timing of Metformin and Radiation

**Figure 1:**
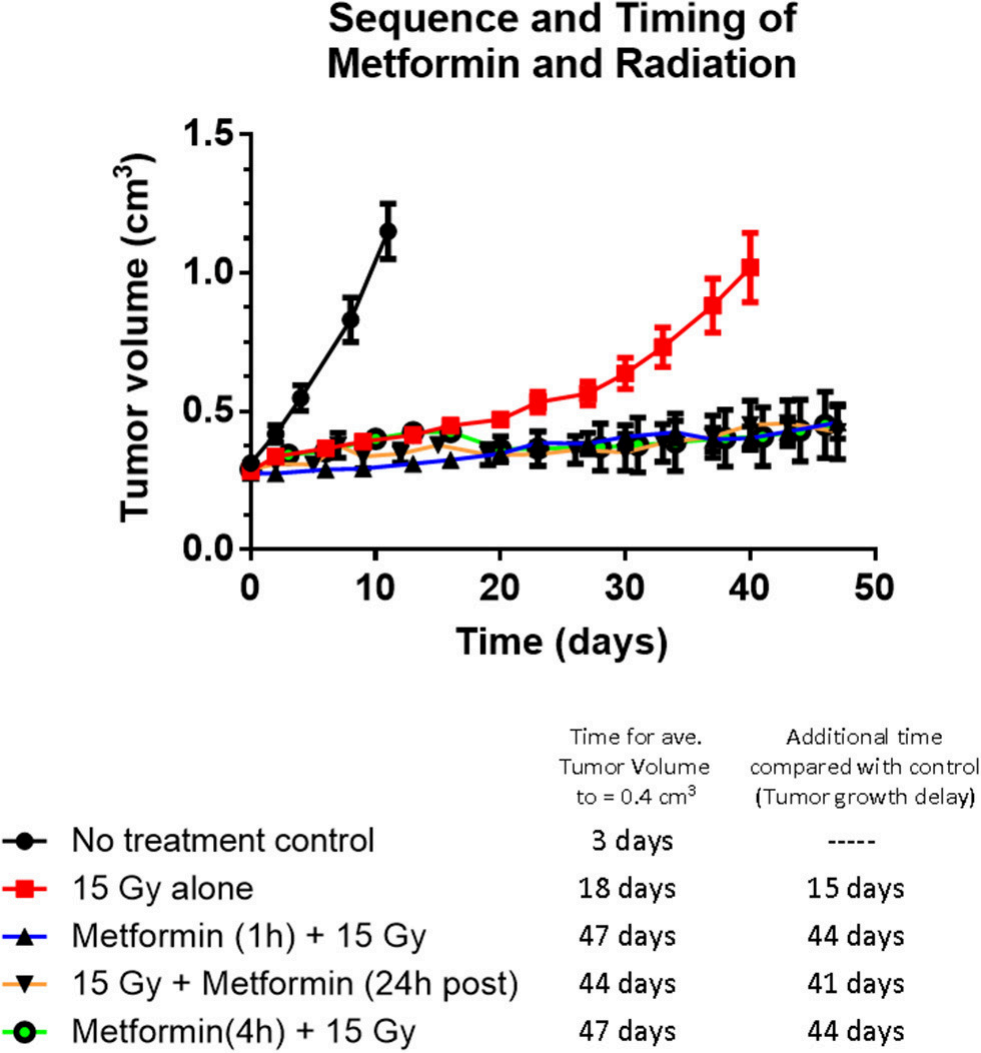
The effect of sequence and timing of metformin and radiation on tumor growth. Tumor growth of A549 human lung carcinoma and tumor growth delay following radiation alone or various timing and sequences of combined administration of metformin (200 mg/kg) and radiation (15 Gy). No difference in tumor growth delay was evident whether metformin was administered 1 or 4 h before radiation or metformin was given 24 h after radiation (all gave growth delays of 43 ± 2 days) indicating the mechanism of radiosensitization cannot be completely explained by metformin's effect on tumor oxygen availability. Symbols represent: Black solid circles are no treatment control tumors (*n* = 14), red squares are 15 Gy alone (n-14), black triangles with blue line are metformin given 1 h before 15 Gy (*n* = 19), open black circles with purple line are metformin given 4 h before 15 Gy (*n* = 4) and upsidedown triangles with orange line are metformin given 24 h after 15 Gy (*n* = 4). The growth of tumors receiving metformin alone was not significantly different to that of no treatment control tumors (data not shown). Error bars represent SEM.

Tumor growth delay studies showed for the first time that metformin administered 1 h before a single large dose of radiotherapy, increased the radiation response (see Figure 1). Radiosensitization by metformin was attributed to improved tumor oxygenation due to metformin-induced mitochondria respiration inhibition. Other drug-radiation timing was examined. When metformin was administered 24 h after radiotherapy, a similar magnitude of radiosensitization was observed (see Figure 1). Consequently, the combined treatment of metformin and radiation either 1 h before or 24 h after irradiation resulted in substantially increased tumor growth delay, *p* < 0.05, compared to metformin alone (not statistically different than no treatment control) or radiation alone (see Figure 1). The radiosensitizing effect of metformin was reduced when metformin was given 72 h after radiation (*p* < 0.05 compared with other timings studied; see Figure S1). These data suggest that the underlying mechanism of radiosensitization by metformin may be derived not only from improved oxygenation of the hypoxic tumor but also from additional cellular mechanisms.

### Extensive Tumor Necrosis When Metformin Follows Radiotherapy

**Figure 2:**
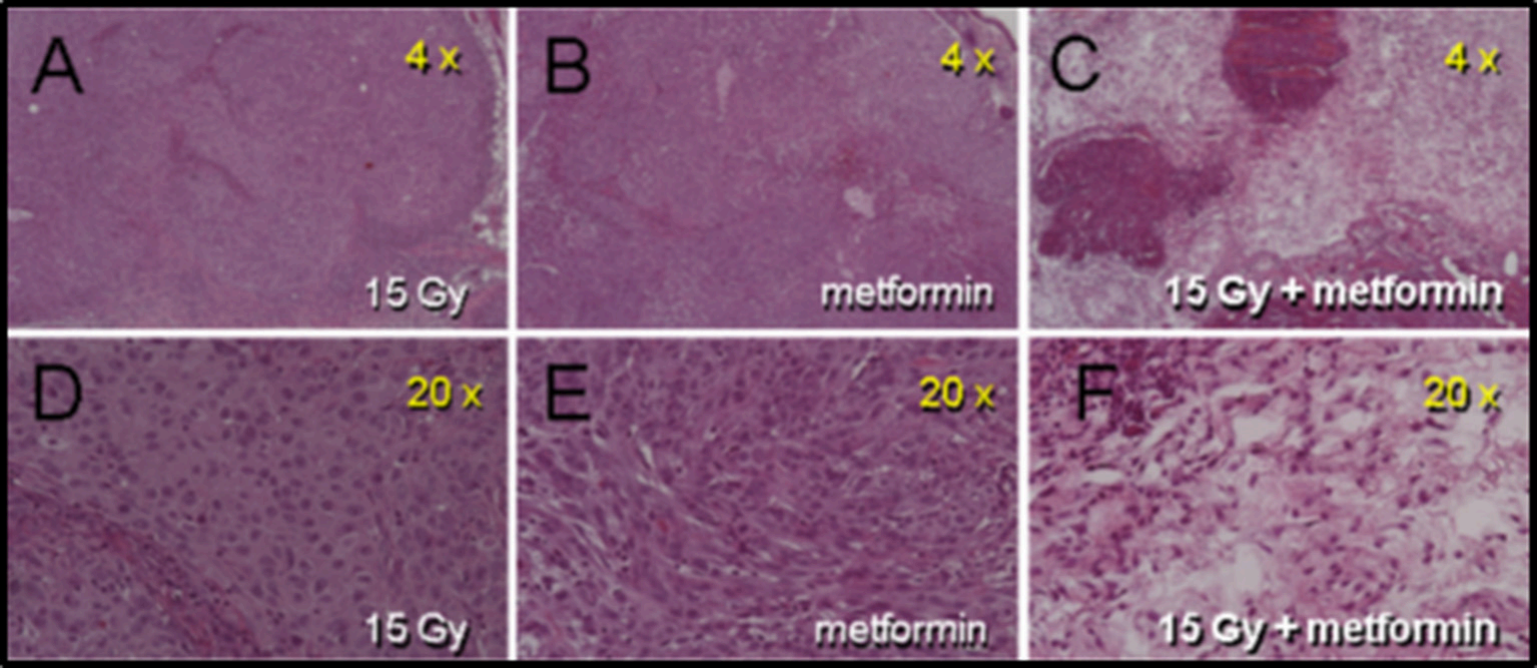
Histologic damage seen in intra-dermally (i.d.) grown A549 tumors receiving metformin 1 h after 15 Gy. Panels **(A–C)** are from mice receiving 15 Gy, metformin (200 mg/kg) and combined radiation and drug, respectively. Panels **(D–F)** are the same tumor sections at higher magnification. Tumors were removed 1 h after metformin (2 h after radiation). Only tumors from mice receiving the combination of drug and radiation show frank damage.

Rapid massive coagulative necrosis occurred in A549 tumors administered metformin after high dose radiation. Figure 2 (intradermally implanted A549 tumors) illustrates extensive necrosis 1 h following combined treatment compared with either radiation alone or metformin alone. Similarly, when tumors were excised 4, 24, or 72 h after the combined radiation plus metformin regimen, extensive necrosis was evident accounting for 50 to 70% of the tumor mass. Figure S2 (previously and sub-curatively irradiated intramuscularly grown A549 tumors) also showed differences in necrosis patterns following subsequent metformin treatment compared with previous radiation alone (~6 weeks before). Previously irradiated tumors showed some signs of damage as seen on H&E sections at low and high magnification (Panel A and C, respectively). There was extensive pimonidazole staining that revealed hypoxia throughout the tumor (~50%) (high magnification example shown in Panel B). In sharp contrast, combined radiation and metformin treated tumors showed extensive damage particularly in the center of the tumor on H&E stained sections (Panel D and F). The central necrosis in the mice with tumors receiving combined drug and radiation was coincident with an absence of pimonidazole staining suggesting non-metabolic, anoxic cells in a poorly perfused tumor fraction that was preferentially killed. Note that in the same tumors, the periphery of the tumor, which appears to be better perfused, showed viable tumor on H&E.

### High Dose Radiation Causes a Rapid Decrease in Tumor Blood Flow

Changes in the tumor blood flow following the combined treatment were measured using an isotope tracer clearance technique. Two mice were studied per group. The blood flow changes were measured starting 1 h after therapy, either radiation alone or combined radiation plus metformin. When combined, radiation preceded metformin by 1 h and tracer clearance started 1 h after metformin administration.

**Figure 3:**
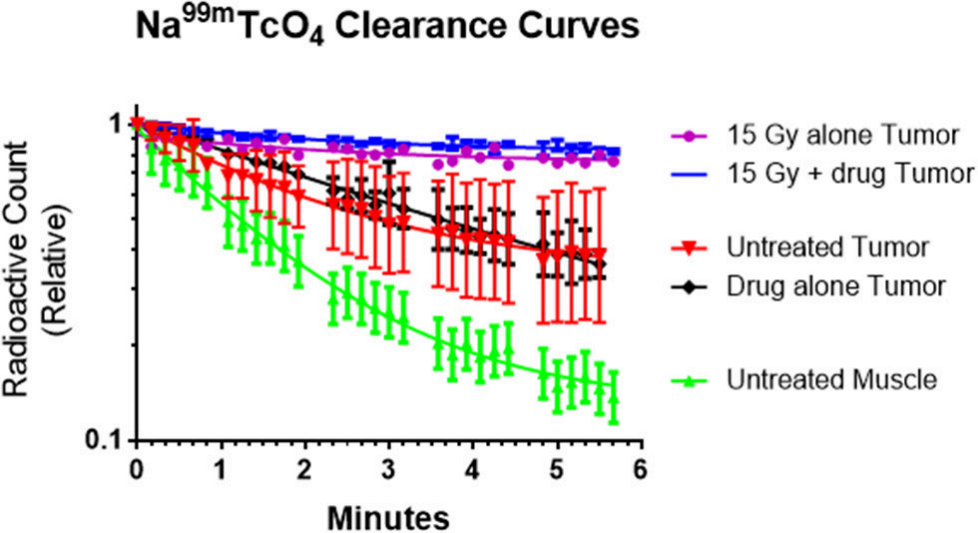
The rate of sodium pertechnetate (Na^99m^TcO_4_) clearance following a local injection was used as a surrogate of blood flow. The radiotracer cleared slowly after high single dose radiation (15 Gy) with or without metformin indicating that the radiation reduced tumor blood flow within an hour of exposure. We hypothesize that decreased blood flow adversely affected tumor microenvironment and enhanced cytotoxicity of metformin. In contrast, radiotracer cleared rapidly from untreated tumors, drug alone tumors and especially normal muscle (control study). Average values (*n* = 2), error bars representing standard deviations and best fit lines assuming a single exponential decay are shown.

Figure 3 presents tumor blood flow changes from untreated tumors and those receiving metformin, radiation or combined therapies 1 h earlier. Normal clearance was observed in control and metformin alone mice. Impaired tumor blood flow was observed in tumors after radiation exposure and combined therapies.

The interpretation of the data is that the effect of radiation with or without metformin on tracer clearance is the same because the radiation causes the blood flow changes. The presence of metformin causes no additional blood flow change.

### Cytotoxicity of Cancer Cells Under Hypoxic and Low Glucose Environment

**Figure 4:**
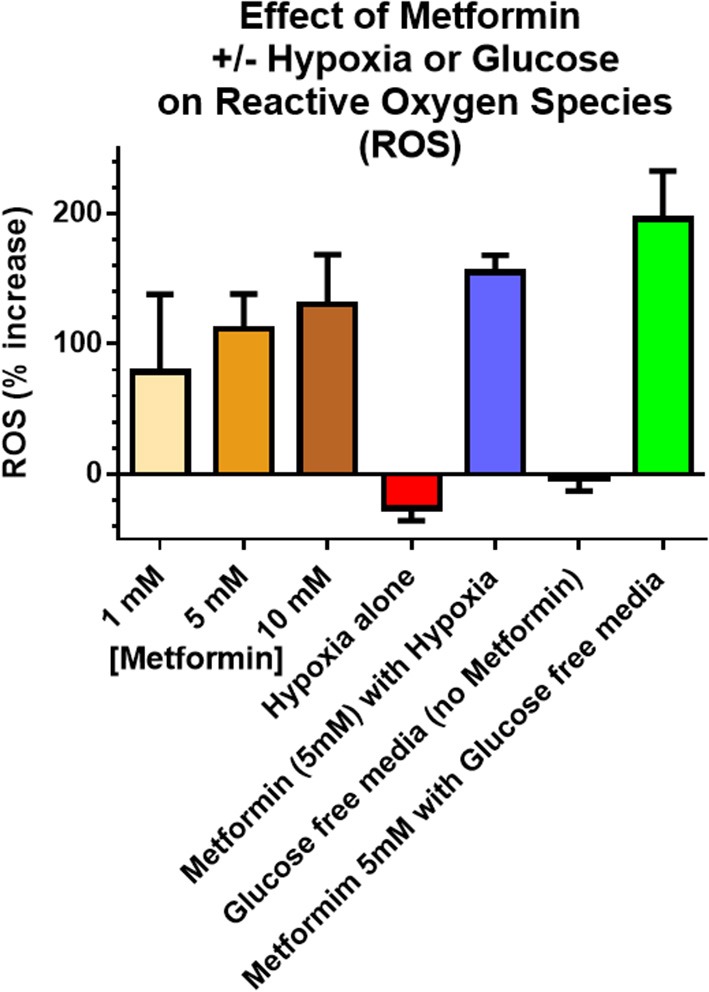
Reactive Oxygen Species (ROS) were used as a surrogate measure of cell killing. Incubation times were 6 h including metformin exposures, glucose concentration and hypoxia. *in vitro* ROS production increased with increasing metformin dose (4.5 g/L glucose), as well as combined hypoxia + metformin (4.5 g/L glucose). The largest increase in ROS occurred after combined glucose free media + metformin. Unless otherwise noted, metformin dose was 5 mM. “Glucose free” indicates an absence of glucose in the media and “Hypoxia” refers to 6 h of 5% CO_2_ plus 95% N_2_ exposure. Error bars represent standard deviations.

ROS production assessed using the fluorescence Dihydroethidium (DHE) was used as a surrogate of cell damage. Enhanced ROS production was observed in cancer cells treated with 5 mM metformin for 6 h under a glucose “free” condition, 0 mM, vs. ROS from cells treated with the same metformin dose under normal glucose conditions, 5 mM glucose. Figure 4 shows the fold increase in fluorescence measured ROS levels in A549 cancer cells in exponential growth phase exposed to graded doses of metformin 1, 5, and 10 mM compare to no metformin (under conditions of 4.5 g/L glucose), and the effect of metformin on ROS under conditions of hypoxia or glucose free media. Similar results were seen with MCF-7 [presented previously (21), MCF-7 produced the same trends but smaller % increases]. It is of note that ROS was significantly higher (*p* < 0.05) under conditions of 5 mM metformin and glucose free conditions compared with 5 mM metformin and high glucose conditions.

**Figure 5:**
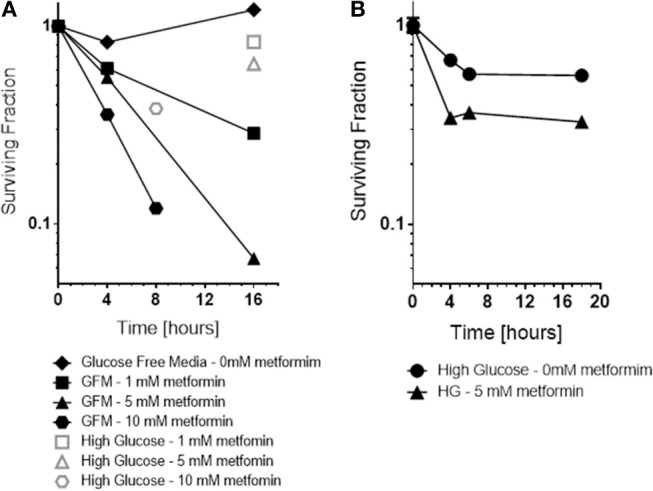
*In vitro*, the cytotoxicity and radiation sensitization of metformin were examined under various microenvironment conditions including glucose concentration **(A)**, pH (not shown because effect size was small) and hypoxia **(B)**. Glucose concentration had the greatest effect; glucose free media increased metformin cytotoxicity and radiation sensitization. For comparison, cell surviving fraction is shown (Open symbols) for cells exposed to 5 mM metformin under “high glucose” conditions.

Consistent with the ROS results, a dramatic increase in cytotoxicity of cancer cells to metformin was observed as a function of exposure time and drug dose under glucose free conditions, 0 mM glucose (see Figure 5). Glucose free media alone for 6 h or even 16 h exposure was not toxic. In contrast, cytotoxicity by metformin increased in a dose and time-dependent manner when the media was deprived of glucose, 0 mM. For example, either 10 mM metformin at 8 h of glucose-deprivation or 5 mM metformin at 16 h of glucose deprivation reduced cell survival by about 1 log, i.e., to 10% of control levels.

Also, as expected based on the ROS results, metformin was more toxic to cancer cells under hypoxic conditions than under oxic conditions (see Figure 5). Metformin alone under oxic conditions exhibited mild cytotoxicity, reducing surviving fraction to 60–70% of control levels. Metformin under hypoxic conditions was significantly more cytotoxic, reducing surviving fraction to 30–40% of control levels.

## Discussion

The results presented are significant for several reasons. The data clearly show that a single dose of metformin is sufficient to produce significant radiosensitization. A single metformin dose combined with high dose irradiation resulted in significant radiosensitization using A549 human tumor xenografts in CD-1 nu/nu mice (Figure 1). The data also demonstrate that the impressive radiosensitization by metformin achieved when metformin precedes high dose irradiation also is realized when metformin follows radiation; our results demonstrate significant radiosensitization when drug is given between 1 h (Figure 2) and 1 day after radiation (Figure 1). Finally, the results illustrate the dependence of timing and sequence on the tumor microenvironment, particularly the glucose concentration and to a lesser extent, the oxygen status (Figures 4, 5). These results complement the current understanding of the mechanism of radiosensitization by metformin.

Metformin pharmacokinetics in mice after intraperitoneal injection has been studied by others (22). Maximal levels of Metformin are reached in plasma and tumor 30 min post-injection with a quarter of the drug maximum remaining after 1 h.

Our data suggest that the underlying mechanism of radiosensitization by metformin may derive not only from re-oxygenation of the existing hypoxic tumor but other microenvironmental considerations that are operative for the enhancement, particularly glucose levels. Mechanistic studies to date have demonstrated that metformin enhances the therapeutic gain of radiotherapy in multiple ways. Song et al. have shown the enhanced radiosensitivity of human breast cancer cells, MCF-7, using both *in vitro* and *in vivo* system (1, 7). They further showed that radiation and metformin together activated AMPK leading to inactivation of mTOR and suppression of its downstream effectors such as S6K1 and 4EBP1. Suppression of 4EBP1 protein leading to reduction in eIF4E enhanced the radiosensitivity of several human cancer cells (23). Others suggest that metformin reduces tumor hypoxia and would be beneficial if administered prior to single dose radiotherapy (8). Our data provide another novel insight into metformin induced radiosensization. Metformin appears to cause selective necrosis in regions of tumor with compromised microenvironment (Figure 2) which is dramatically enhanced by a marked decrease in tumor blood flow observed after large single radiation exposures (Figure 3).

Tumor hypoxia has an important influence on the outcome of therapy. Many large sized rodent and human tumors contain varying fractions of highly radioresistant hypoxic tumor cells. Equally important relative to radiosurgery, i.e., single high dose irradiation, is the fact that the hypoxic fraction increases following a single high dose of radiation due in part to tumor vascular collapse (Figure 3). Song et al. also have shown that the tumor hypoxic fraction significantly increases following radiosurgery in their murine models (24, 25). Further, they have shown that HIF-1 alpha concomitantly increases. Several compounds have been shown to suppress HIF-1 alpha including metformin (26–28). However, the role of the inhibitory effect of metformin on HIF-1 alpha in the present study was not explored. Similarly, it is not clear whether mitochondrial inhibitors such as metformin and arsenic trioxide would preferentially kill radiation-induced tumor hypoxia (29–32).

Current on-going clinical trials are investigating the combined use of daily low-dose metformin with fractionated radiotherapy. Irrespective the underlying mechanism of radiation enhancement by metformin, the present data provide a pre-clinical rationale of combining radiosurgery and single high dose of metformin in the clinical settings.

## Conclusion

Radiosensitization by metformin has been demonstrated previously and attributed to the PI3K/AKT/mTOR pathway. Others proposed that metformin improves radiosensitization through improved oxygenation (2). In the current study, results support a possible third mechanism of radiosensitization by metformin based on the microenvironment, particularly glucose concentration at the time of metformin administration. Single high-dose radiation exposure leads to a profound decrease in tumor perfusion that could adversely affect the microenvironment and potentiate metformin cytotoxicity. Our on-going *in vivo* xenograft study indeed shows a preferential killing of cancer cells under conditions consistent with low oxygen and low glucose following a single high-dose of radiation therapy and metformin. Future work includes exploring (1) the optimum timing under fractionated high-dose radiation therapy, and (2) alternative strategies to reduce glucose in the microenvironment prior to metformin administration. Finally, the results of the current study have help explain the response of high dose radiotherapy used clinically for cancers of the brain (SRS) and other sites (SBRT), when cancer patients also have diabetes and are taking metformin.

## Author Contributions

JK conceived the presented idea. JK, SB, AK, DI, KA, KL, and KJ planned the studies. AK, SB, DI, KA, KL, and KJ carried out the studies. JK, SB, AK, DI, KA, KL, and KJ contributed to interpretation of the results. JK and SB took the lead in writing the manuscript. JK, SB, AK, DI, KA, KL, and KJ provided critical feedback and helped shape the research, analysis and manuscript.

### Conflict of Interest Statement

The authors declare that the research was conducted in the absence of any commercial or financial relationships that could be construed as a potential conflict of interest.

## Supplementary Material

Figure S1 The radiosensitizing effect of metformin when given 72 h after radiation (green circle symbols) was significantly less (*p* < 0.05) than that of metformin given 1 h after radiation (triangle symbols), however the radiosensitizing effect remained significant (*p* < 0.05) compared to radiation alone (red square symbols).

Figure S2 Tumors receiving metformin after radiation exhibit extensive gross damage and large regions void of pimonidazole staining unlike untreated control tumors and those administered metformin alone. A549 intramuscular tumors previously treated with 15 Gy, allowed to regrow for approximately 6 weeks and then either harvested **(A–C)** or treated with metformin (200 mg/kg) **(D–F)** and harvested 2 h later. Yellow arrows in Panel E illustrate regions void of pimonidazole.
