# Supplementary figures and images for: A Novel Mechanism of High Dose Radiation Sensitization by Metformin
Source: Front Oncol. 2019 Apr 9;9:247. doi: 10.3389/fonc.2019.00247 (PMC6465931; doi:10.3389/fonc.2019.00247)

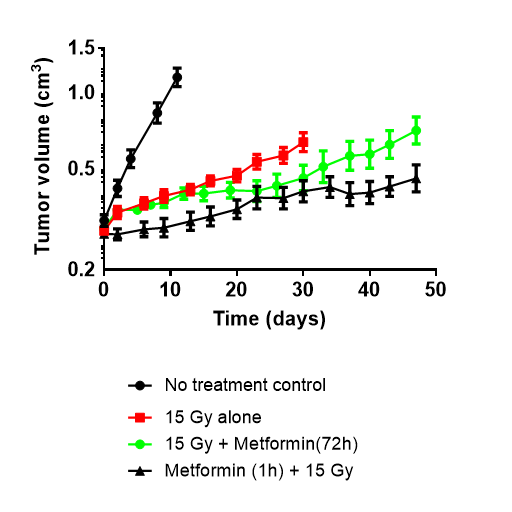

Supplement: Figure S1 — The radiosensitizing effect of metformin when given 72 h after radiation (green circle symbols) was significantly less (p < 0.05) than that of metformin given 1 h after radiation (triangle symbols), however the radiosensitizing effect remained significant (p < 0.05) compared to radiation alone (red square symbols). [file Image_1.TIF]

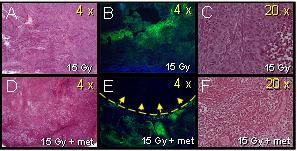

Supplement: Figure S2 — Tumors receiving metformin after radiation exhibit extensive gross damage and large regions void of pimonidazole staining unlike untreated control tumors and those administered metformin alone. A549 intramuscular tumors previously treated with 15 Gy, allowed to regrow for approximately 6 weeks and then either harvested (A–C) or treated with metformin (200 mg/kg) (D–F) and harvested 2 h later. Yellow arrows in Panel E illustrate regions void of pimonidazole. [file Image_2.TIF]
